# Study Protocol - Alcohol Management Plans (AMPs) in remote indigenous communities in Queensland: their impacts on injury, violence, health and social indicators and their cost-effectiveness

**DOI:** 10.1186/1471-2458-14-15

**Published:** 2014-01-09

**Authors:** Alan R Clough, Michelle S Fitts, Jan A Robertson, Anthony Shakeshaft, Adrian Miller, Christopher M Doran, Reinhold Muller, Valmae Ypinazar, David Martin, Robyn McDermott, Rob Sanson-Fisher, Simon Towle, Stephen A Margolis, Caryn West

**Affiliations:** 1 Community-based Health Promotion and Prevention Studies Group, Australian Institute of Tropical Health and Medicine, James Cook University (Cairns Campus), PO Box 6811, Cairns, Qld 4870, Australia; 2 Centre for Accident Research and Road Safety - Queensland (CARRS-Q), Queensland University of Technology, Brisbane, Qld 4059, Australia; 3 School of Public Health, Tropical Medicine & Rehabilitation Sciences, School of Nursing, Nutrition & Midwifery, James Cook University, PO Box 6811, Cairns, Qld 4870, Australia; 4 National Drug and Alcohol Research centre, University of NSW, Sydney, NSW 2052, Australia; 5 Indigenous Health, Indigenous Research Network, Griffith University, Brisbane, Qld 4111, Australia; 6 Hunter Medical Research Institute, University of Newcastle, Locked Bag 1000, New Lambton Heights, NSW 2305, Australia; 7 School of Public Health, Tropical Medicine and Rehabilitation Sciences, James Cook University (Cairns Campus), PO Box 6811, Cairns, Qld 4870, Australia; 8 Adjunct Senior Research Fellow School of Medicine and Dentistry, James Cook University, Cairns 4870, Australia; 9 ANU College of Arts and Social Sciences, Acton, ACT 0200, Australia; 10 Centre of Research Excellence for Preventable Chronic Conditions, Australian Institute for Tropical Health and Medicine, James Cook University, PO Box 6811, Cairns, QLD 4870, Australia; 11 Health Behaviour, Health Behaviour Research Group, School of Medicine and Public Health, University of Newcastle, Newcastle, Australia; 12 Department of Aboriginal and Torres Strait Islander and Multicultural Affairs, Level 2, William McCormack Place, 5B Sheridan Street, PO Box 5365, Cairns, Queensland 4870, Australia; 13 School of Medicine, Griffith University, Parklands Drive, Southport, Queensland 4215, Australia; 14 School of Nursing, Midwifery and Nutrition, James Cook University, Po Box 6811, Cairns, QLD 4870, Australia

**Keywords:** Alcohol, Indigenous, Alcohol Management Plans, Public Health

## Abstract

**Background:**

In 2002/03 the Queensland Government responded to high rates of alcohol-related harm in discrete Indigenous communities by implementing alcohol management plans (AMPs), designed to include supply and harm reduction and treatment measures. Tighter alcohol supply and carriage restrictions followed in 2008 following indications of reductions in violence and injury. Despite the plans being in place for over a decade, no comprehensive independent review has assessed to what level the designed aims were achieved and what effect the plans have had on Indigenous community residents and service providers. This study will describe the long-term impacts on important health, economic and social outcomes of Queensland’s AMPs.

**Methods/Design:**

The project has two main studies, 1) outcome evaluation using de-identified epidemiological data on injury, violence and other health and social indicators for across Queensland, including de-identified databases compiled from relevant routinely-available administrative data sets, and 2) a process evaluation to map the nature, timing and content of intervention components targeting alcohol. Process evaluation will also be used to assess the fidelity with which the designed intervention components have been implemented, their uptake and community responses to them and their perceived impacts on alcohol supply and consumption, injury, violence and community health. Interviews and focus groups with Indigenous residents and service providers will be used. The study will be conducted in all 24 of Queensland’s Indigenous communities affected by alcohol management plans.

**Discussion:**

This evaluation will report on the impacts of the original aims for AMPs, what impact they have had on Indigenous residents and service providers. A central outcome will be the establishment of relevant databases describing the parameters of the changes seen. This will permit comprehensive and rigorous surveillance systems to be put in place and provided to communities empowering them with the best credible evidence to judge future policy and program requirements for themselves. The project will inform impending alcohol policy and program adjustments in Queensland and other Australian jurisdictions.

The project has been approved by the James Cook University Human Research Ethics Committee (approval number H4967 & H5241).

## Background

Alcohol Management Plans (AMPs) were first implemented by the Queensland Government over a decade ago in 2002-03 [1]. More recently, in 2008, alcohol was further prohibited locally in several Indigenous communities and restrictions on quantity and type of alcohol that could be legitimately possessed in these communities were reinforced. The Queensland Government currently has AMPs under review [2] bringing the prospect that alcohol may become readily available once more in some localities.

Currently a focus for Australian Government action generally, improving health in the discrete Indigenous communities in rural and remote Queensland has arisen as a particular concern, especially since the 1990s [3-7]. Closely linked to Indigenous health outcomes is the effect of alcohol-related violence and injury which is an ongoing concern in Indigenous communities across Australia [3,8-14]. From 2002–03, the Queensland Government responded to these issues with a range of strategies to reduce alcohol misuse, violence and injury in communities [1,3-6,8-14]. These strategies appear to have had some positive effects [13,15,16] for example from 2002; hospital admissions for assault decreased in eight of the 17 discrete Indigenous communities where strategies were first implemented [5]. However, offences against the person fell in just five communities [5] suggesting varying impacts. The most recent Queensland Government key indicators report (January - March, 2012) suggests only two communities have shown both a decline in hospital admissions for assault and a decline in offences against the person [17]. For four communities in Cape York, data for the period 1995–2005 describes Royal Flying Doctor Service (RFDS) aero-medical retrievals for serious injury, many linked with alcohol misuse [4,14]. Rates of retrieval were initially halved after alcohol supply controls were implemented in 2002–03, but appeared to rise again two years later in 2004 [4]. For the same four communities, further supply control strategies implemented at the end of 2008 may have reinforced an overall downward trend in RFDS emergency retrievals in these four communities to historically low levels [7]. If there have been similar reductions in other communities, strategies to sustain such important changes are needed.

However, Queensland’s strategies to reduce this kind of alcohol-related violence and injury have been very complex in their nature, timing and content, and have not been rigorously evaluated. Possible shifts in alcohol supply and consumption have been ignored as has a possible increase in other substance misuse in these communities. AMP design has varied across the communities and implementation appears piecemeal with possible successes in some localities not sustained or transferred to others. Community responses seem to have also varied since 2002–03. Clearly, for sustainable strategies, there must be sound evidence to design more robust policy into the future [6,15,16] along with a more rigorous evaluation framework [18,19].

Heralding a new policy phase in this increasingly complex set of reforms, from early 2011, the former Queensland Government commenced to embed harm reduction targets into ‘Community Safety Plans’ in AMP communities consistent with its new ‘Strong Community Life’ approach [20] and Just Futures Strategy [21]. Unpublished consultations and observations indicate that this new policy approach was widely resented and opposed in many of the communities. Under the ‘Community Safety Plans’ policy, targets were set by the Queensland Government’s Department of Justice. Each community became benchmarked against regional estimates of hospital admissions due to assault and numbers of offences-against-the-person reported to police. Not surprisingly, community Councils have opposed this benchmarking arguing that hospital admissions and violent incidents are often multiple incidents perpetrated by just a few individuals or the incidents relate to individuals not normally resident in their communities. Whether this is backed by empirical evidence, and however flawed or misleading the Queensland Government indicators may have been, in 2011, the former Government advised community leaders through a peak local government body that it was seeking an ‘exit strategy’ from AMPs, promising to review restrictions if the targeted reductions in harm indicators could be reached and sustained. It is not yet clear how the approach the new Queensland Government (elected March 2012) is different except that it has devolved responsibility for any modifications to AMPs to each affected community [2]. With the limited and equivocal information available to inform a review of these historically important policies at this critical juncture, the need for systematic research is particularly urgent. Successful strategies of crucial importance to many Indigenous Australians may not be fully understood, and important lessons learned may be lost in further ‘policy drift’ if both effective and ineffective strategies are not systematically documented.

Against this background of evolving policies, programs and changing Governments, there has been no comprehensive, systematic evaluation of the impacts and effectiveness of these extraordinarily complex alcohol controls in Queensland. Much relevant information for these settings is either unpublished or is in community-specific evaluations or administrative data not subjected to rigorous analysis. Although there may have been a reduction in alcohol problems, very little is known about impacts on alcohol availability and consumption, on health, and the wider and longer-term implications for individuals, families and communities. Our study thus provides a timely, rigorous and objective outcome and process evaluation along with a cost-effectiveness assessment for future evidence-based policies in Queensland. The evaluation framework used is likely to be applicable in other Australian jurisdictions where alcohol and violence in Indigenous communities is the focus of national policy and program attention.

### Aim

This study aims to describe the long-term impacts on important health, economic and social outcomes of complex interventions restricting the supply of alcohol in Indigenous communities in Queensland. The study will assess impacts of past policy shifts, program changes and local responses to the Queensland Government’s Alcohol Management Plans (AMPs) implemented from 2002–03. It will provide an evaluation framework and evidence base to inform communities and governments if future policy and program adjustments are proposed in these and similar Indigenous communities elsewhere in Australia. Specifically the study aims to:

i) conduct an outcome evaluation using de-identified epidemiological data on injury, violence and other health and social indicators across rural and remote Queensland, including de-identified databases compiled from relevant routinely-available administrative data sets,

ii) conduct a process evaluation to map

a. the nature, timing and content of intervention components targeting alcohol.

a. the fidelity with which intervention components have been implemented, their uptake and community responses to them and their perceived impacts on alcohol supply and consumption, injury, violence and community health, and

iii) assess the cost-effectiveness of these strategies.

## Methods/Design

### Setting & location

**Figure 1:**
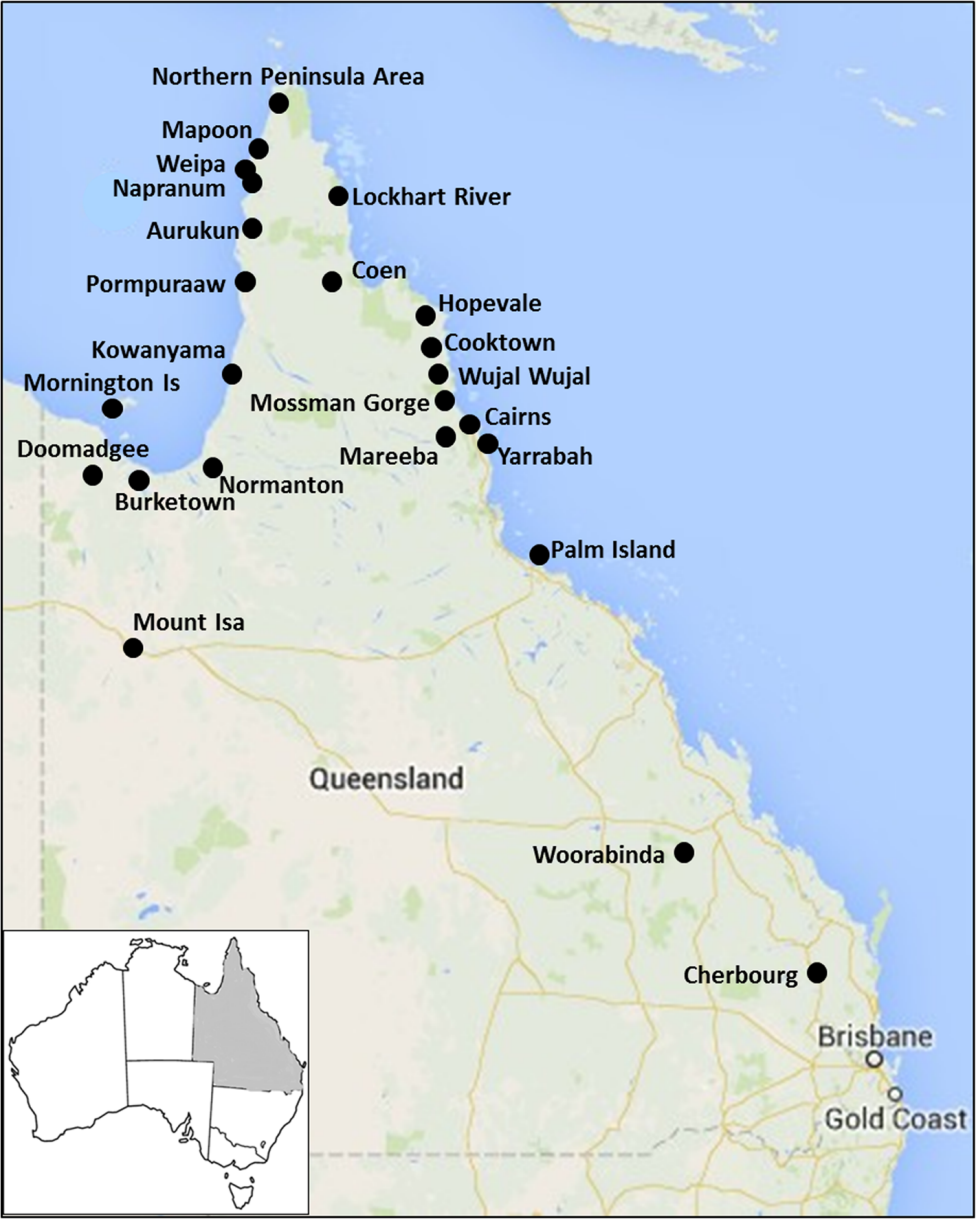
Indigenous communities and towns in Queensland affected by Alcohol Management Plans.

The study will be conducted in 24 of Queensland’s Indigenous communities and in the towns nearby (Figure 1). The Indigenous communities range in size from small localities with around 150 people situated close to regional centres up to large communities of around 3000 people. Regional boundaries for compiling health and administrative data vary considerably across Queensland Government agencies, an issue which the proposed study will address.

### Participants and recruitment

The estimated Indigenous populations directly affected by Alcohol Management Plans in the study communities were approximately 16000 at the 2011 census. These communities include around 600 non-Indigenous residents who live and work in the communities. The regional towns nearby the directly-affected communities have more mixed populations of from 100–3500 people but with substantial proportions of Indigenous residents. Service providers and visitors from the major regional centres along with visitors from other parts of Queensland and Australia are also potentially affected. The study population is defined as the usual resident population of the 24 communities and the nearby towns as at the 2011 census but also includes relevant service providers and key stakeholders who live and work in the main regional centres.

**Figure 2:**
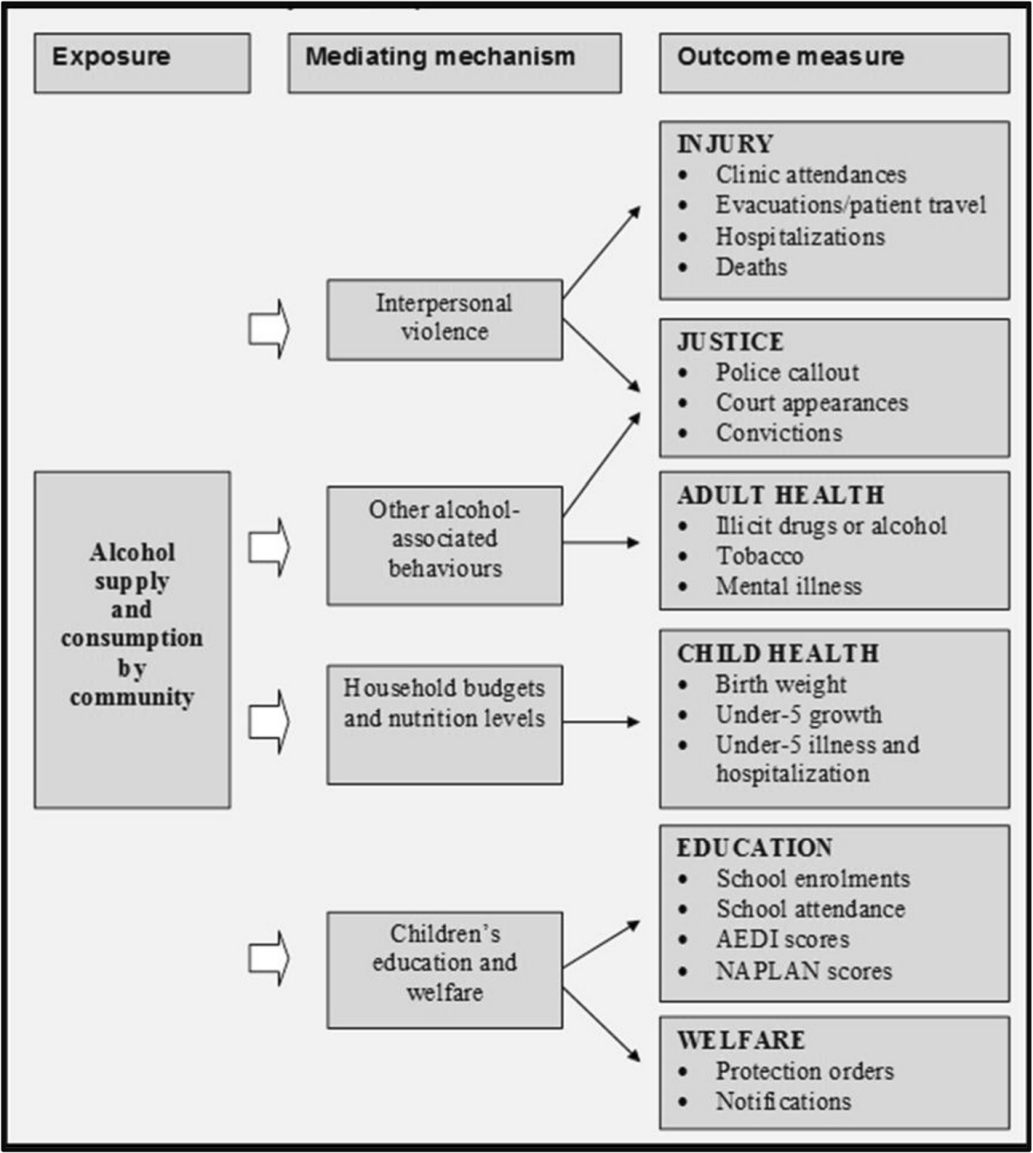
AMP study logic model.

i) Outcome evaluation: Figure 2 outlines the quantitative data the study aims to examine. This data will be compiled for Indigenous people normally resident in the affected Indigenous communities and in the nearby towns.

ii) Process evaluation: Participants from stakeholder and service agency groups (Table 1) will be recruited for formal interviews or focus group discussions. There are no exclusion criteria except that those interviewed will ideally have direct experience of the AMPs from the beginning of Phase 1, from 2001. At least two participants will be interviewed in each subcategory, making a total of at least 76 participants. A balance in the sample between Indigenous and non-Indigenous participants and between the frontline stakeholders at the community level and those who are removed from frontline service provision will be sought.

**Table 1:** Stakeholder groups and service agencies to be interviewed in process evaluation

**Stakeholder group or service agency**		**Community-based**	**Regionally based & visiting community**	**Service providers in region**
**Health**				
	General	I		
	Drug & alcohol workers		I	
	Mental health workers		I	I
	Residential rehabilitation services			I
**Justice and liquor regulation**				
	Queensland police service	I	I	I
	Legal services		I	I
	Magistrates			I
	Community justice groups	I		
	Family responsibilities commission	I	I	I
	Liquor accords	I	I	I
**Education**				
	Managers and principals	I	I	
	Employees	I		
**Welfare**				
	Child safety	I	I	
	**Local government councils and community members**			
	Elected councillors	I		
	CEOs and managers	I		
	CDEP	I		
	Night patrol	I		
	Community members	I		
	Men’s groups	I		
	Women's groups	I		
	**Non-government organisations**			
	RFDS		I	
	Cape York Partnerships		I	I
	Apunipima Cape York Health Council	I	I	I
	Employment groups	I	I	

### Funding

Funding for the study was provided by the National Health and Medical Research Council of Australia (NHMRC, #APP1042532 project grant).

### Ethics

The study was approved by the Human Research Ethics Committee James Cook University (H4967 & H5241). The project was considered and supported by the Indigenous Leaders Forum of the Local Government Association of Queensland which included the duly elected Mayors and CEOs from all affected Queensland communities. Approval was also sought from the following Human Research Ethics Committees: Cairns and Hinterland Health Services District Human Research Ethics Committee and Townsville and District Human Research Ethics Committee.

### Staff

Supporting the Chief Investigators, two research fellows were initially employed as three year, part-time post-doctoral fellows for the course of the study. Two project officers and four casual research assistants were also employed. Of these, one is an Indigenous person. Additional people can be employed on a casual basis during visits to discrete communities; these community members act as local facilitators within the community. The majority of staff members have prior experience and qualifications working with Indigenous community members or in remote Indigenous communities in Queensland and in other Australian jurisdictions.

### Consent

Potential participants are all provided with written information about the study and a face-to-face discussion is held with the participant given the opportunity to ask questions. If required, an interpreter can be employed to explain all information relating to the study. Those indicating that they wish to participate are then asked to complete and sign a consent form. Consent is obtained using the NHMRC Guidelines for Ethical Conduct in Aboriginal and Torres Strait Islander Health Research. All participants are informed that their participation is voluntary and that they can refuse or withdraw from the study without the need to provide reasons or justification for their decision. Participants are asked to give separate consent for various elements of the study, including: the use of an audio recording device, permission to be photographed or participate in a focus group.

### Study overview and choice of study designs

#### ***Outcome evaluation study design: ecological studies***

**Figure 3:**
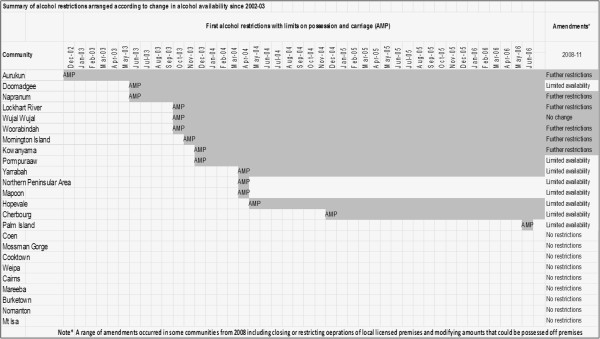
Communities and towns arranged according to change in alcohol availability.

The outcome evaluation design is based on mainly ecological studies. An ecological approach facilitates the compilation of injury, violence, health and wellbeing data in a regional framework for future surveillance. The core of the evaluation design incorporates quasi-experimental, interrupted time-series analyses of outcome indicators for injury, violence against the person and major health and social factors across the 24 study communities. Comparisons will be made between communities with tighter alcohol restrictions and those where there has been little change or no change, matched as far as practicable. In Figure 3, the 24 study communities and towns are arranged in order of the first implementation of AMPs. Note that in two communities no restrictions on alcohol availability were initially imposed while in others the timing of the initial imposition of possession and carriage is staggered over 18 months from December 2002 through to May 2004 (Figure 3). A number of licensed outlets continue to be run by private enterprise in some localities and in six communities, Council-run ‘canteens’ operated to the end of 2008 providing alcohol locally to community residents. In 2008, there were further abrupt restrictions in the local availability of alcohol when all but one of these six Council-run, community ‘canteens’ were closed, with tight restrictions on serving times in the one canteen permitted to continue (Figure 3). The staggered introduction of AMPs, and the abrupt changes in alcohol availability in some communities and not in others, provides the unique opportunity to assess the impacts of alcohol-restrictions in a quasi-experimental fashion using an interrupted time-series, multiple baseline approach.

#### ***Outcome evaluation data***

Government and non-government service agencies collect a range of administrative and financial data held in departmental collections or locally. Figure 2, Logic Model, outlines the data the study aims to examine. The core data will include Queensland Health data including: clinical activity, evacuations, emergency retrievals by the Royal Flying Doctor Service, patient travel, hospital separations, deaths, birth weights, child nutrition, and notifications for selected conditions. Justice and Corrections data: court rolls, convictions and imprisonment data and Adult Health Check and Child Health information. The study will also seek to compile Education Queensland data on enrolments, Australian Early Development Index, National Assessment Program – Literacy and Numeracy and Department of Communities’ information on child protection notifications. Data will be de-identified but with a unique project ID code to allow us to distinguish multiple service usage by individuals. As administrative boundaries overlap to varying degrees, all data will be geo-coded to allow for analysis at community and regional levels, and the calculation of rates for the communities in each region. Denominator populations will be derived from Australian Bureau of Statistics estimates by interpolation. This study will not follow or create cohorts; rather an ecological approach will be taken which will map changes in rates of outcomes over the study period. This can then be mapped against descriptive data of community-level interventions obtained in the process evaluation which will provide trends over time.

#### ***Process evaluation design***

The process evaluation will include document review, interviews and focus groups. The fidelity with which intervention components are implemented, their nature, timing and content and community responses will be of high importance. Process and policy questions will include:

1. How have the communities and their support organisations engaged with and responded to alcohol restrictions?

2. Are there any unintended consequences? What are they and how have these been addressed?

3. What have been the impacts on individuals’ drinking and other substance use patterns and on people’s health and wellbeing?

4. Has there been significant migration from the affected communities to localities where alcohol is more readily available and, if so, what are the consequences of this?

5. What implications and challenges are there for the communities and the Queensland Government if alcohol restrictions are maintained in their present form?

### Process evaluation sampling design

Participants from stakeholder and service agency groups (Table 1) will be recruited for formal interviews or focus group discussions. Initially each group or agency will be approached using existing networks. Semi-structured interviews will be performed to ensure we sample exhaustively, and each participant will be asked to recommend others for interview. Those interviewed will ideally have direct experience of the AMPs from the beginning of Phase 1 in 2001. At least two participants will be interviewed in each subcategory, making a total of at least 76 participants. However, the sample size is likely to be considerably larger than this as, using a ‘snowball’ process, we will be recommended to other potential participants by people in the sample. Sampling will continue until saturation is reached, indicated when participants interviewed begin to refer us to the same kinds of agencies or individuals and by preliminary analysis of emergent themes [22]. A balance in the sample between Indigenous and non-Indigenous participants and between the frontline stakeholders at the community level and those who are removed from frontline service provision will be sought.

### Process evaluation plan to assess fidelity of implementation of intervention components

Carroll et al [23] conceptualised the measurement of the degree of *fidelity* of an intervention as potentially influenced by moderating factors reflected in indicators of *adherence* to the original program design [23]. The measurement of *adherence* relates to the *content* and quantity of active ingredients of the intervention and whether they were delivered widely (*coverage)* as often and for as long as was planned (*frequency, intensity and duration)*[23]. The design, framework for data collection and the types of data sources and collection procedures to be used are listed in Table 2.

**Table 2:** The process evaluation plan, process questions, data sources and collection methods

**Topics for measurement**	**Process questions**	**Types of data sources and collection**
**Evaluation of adherence to implementation of alcohol restrictions in Cape York communities**		
Content	Was each of the components implemented as planned in each phase?	Analysis of documents
		Focus groups with stakeholders
Frequency/intensity/duration (dose delivered)	Were the components implemented as often and for as long as planned?	Analysis of documents
		Focus groups with stakeholders
Coverage (reach)	What proportion of the community population were exposed to or participated in the intervention delivery?	Focus groups with stakeholders
**Potential moderating factors**		
Community responsiveness (dose received)	How and to what extent did the community population engage with or circumvent the restrictions?	Analysis of documents
		Focus groups with stakeholders
Intervention complexity	How complex are the restrictions?	Reference group to evaluate
Comprehensiveness of intervention description and policy	How specific is the description of the provisions of the alcohol restrictions?	Reference group to evaluate
Strategies to facilitate implementation	What strategies used and how were they perceived by community populations?	Focus groups with stakeholders
Quality of delivery	Rating of quality	Focus groups with project workers
Recruitment or engagement	What recruitment procedures were used to engage with community members and stakeholder groups?	Focus groups with stakeholders
Context	Did any factors external to the restrictions affect their implementation?	Focus groups with project workers and stakeholders

Potential moderators of intervention *fidelity* include the quality of Queensland’s AMP program design, particularly whether the AMP programs are specified in ways that can be taken up at a local level across cultural boundaries, how it is facilitated locally, the quality of the delivery, the priority placed on the intervention program in the context of other community priorities, and how interested local stakeholders have been to engage with the intervention components. The action research approach underpinning this design adds capacity to provide progressive feedback of study results to communities and to the study’s reference group.

### Cost-effectiveness analysis

A cost-effectiveness analysis will be conducted using the ‘Assessing cost-effectiveness’ (ACE)-Alcohol approach, consistent with the ACE-Prevention methodology. These methods are international best-practice and include: the adoption of a social perspective; transparent and scientific methods to identify, measure and value both costs and outcomes; modelling and uncertainty testing of epidemiological and costing input parameters; and interpretation of results within a broader decision-making framework.

### Data analysis

#### ***Quantitative outcome measures***

Since the number of independent units (communities) is small in statistical terms, multivariate approaches are generally excluded and inferential statistics will be exclusively based on exact versions of standard bivariate test procedures. Exact binomial and Poisson distribution based confidence intervals and comparisons will be employed for the time trends of rates of health and other outcomes as appropriate. The main analytical emphasis in this case, however, will be rather on the logical “triangulation” of results than on reliance on p-values alone. A comparative analysis of time trends in the outcome measures will be conducted. The ability to simultaneously assess different injury, health and other outcomes from independent data sources (hospital separations, community clinic presentations, police data, etc.), over a minimum ten year period across different phases of the AMP interventions in the 24 communities will assist to control potential information bias in analyses. Cross-sectional time-series modelling will be used to compare trends over time with the main analytical emphasis on triangulation of the trends supported by graphical analysis.

#### ***Process evaluation data analysis***

Data analysis will use standard qualitative data analysis procedures with a codebook developed to aid in content analysis of transcripts of interviews and focus groups and to analyse documents. Once verified, the data are coded into topics and links between key concepts and topics mapped. As is standard practice in community-based studies, a project reference group will consider the process evaluation results.

#### ***Cost-effectiveness analysis***

A multi-state life table Markov model will be used to calculate health and economic outcomes resulting from a reduction in alcohol-use due to the AMP. A range of alcohol-related injuries and diseases can be modelled. The ACE model summarises the disease-specific changes in the number of years lived, adjusted for disability from the explicitly modelled diseases and average age and sex-specific disability levels from all other causes [24-26]. The costs and health outcomes are summed over the lifetime to determine the incremental cost-effectiveness ratio (ICER), in dollars per disability adjusted life year (DALY) averted. Monte Carlo analysis is used to derive 95% uncertainty intervals for all outcomes and to determine the probability of intervention cost-effectiveness against a cost-effectiveness threshold of $50,000 per DALY. ICER results are displayed on a cost-effectiveness plane with affordability issues addressed in an acceptability curve. The results of the cost-effectiveness analysis will be considered in the context of other decision making criteria including: strength of evidence; capacity of the intervention to reduce inequity; acceptability to stakeholders; feasibility; sustainability; and, potential for other consequences. This will provide the opportunity to broaden the ACE Alcohol model’s scope, to incorporate non-health consequences, especially person-to-person violence and interactions with the justice system.

### Timeline

The study will be conducted over a minimum three year period which includes time for reporting, consultation with data providers, community groups and policy units. Modifications to the database construction will be performed to ensure sustainable data collection, analysis and reporting of community and regional benchmarking and population level trends.

### Project governance

The project is overseen by a reference group representing potential knowledge brokers and knowledge users, which includes representatives of peak community groups, including elected Local Government Mayors and Councillors, data providers and custodians and chief investigators. To effectively achieve the project objectives (data provision, curation, analysis, reporting, dissemination and turning the results into policy and programs) the reference group will include the following stakeholders: Aboriginal and Torres Strait Islander community-controlled health sector, Community Justice Groups mandated in each community, key Indigenous policy groups, the Queensland Local Government Association’s Indigenous Leaders’ Forum, Queensland Government Departments, including Health, Education and the Queensland Police Service. Inviting the involvement of Queensland’s Department of Communities and the Office of Liquor and Gaming will assist to ensure that results of the study can be used to inform future Queensland Government agencies.

### Outcomes & Significance

Reducing the harms linked with alcohol misuse in remote Indigenous communities has been a challenge for Australian governments and Indigenous leaders for half a century [27-30]. Apparent successes in some localities have been difficult to sustain. Furthermore the successful transfer of approaches beyond the local region or community is very challenging [27,30]. The literature evaluating community-level interventions to address alcohol problems in Indigenous Australian communities is diffuse with little precise information outside of Government reports [17,31,32] or the many community-specific evaluations carried out so far [30,33]. Research in this field lacks a rigorous methodological basis. This study addresses this lack by providing a rigorous, systematic methodology with credible outcome measures and robust cost-effectiveness assessments.

AMPs were implemented in response to what was widely regarded as a public health emergency in some of the communities, with extremely high rates of alcohol-related violence [34] including effects on family and child health and well-being. Observations over subsequent years since their implementation, show there were fragmented attempts to evaluate the impact of AMPs on injury rates, school attendance etc., but with no agreed systematic approach to data collection and reporting. This has led to anecdotal claims of impact or lack of it, by various lobby groups, but with very few of these supported by robust data. There is an urgent need to compile injury, violence and health and wellbeing data at the regional level, a need also addressed by this study. A central outcome will be the establishment of relevant databases describing the parameters of the changes seen. This will permit comprehensive and rigorous surveillance systems to be put in place and provided to communities empowering them with the best credible evidence to judge future policy and program requirements for themselves.

If historically important changes have in fact been achieved, it is vital to understand how successful elements can be transferred to similar regions. The study’s results and methods will be timely to inform impending policy and program adjustments in other Australian jurisdictions. For example, the recently tabled ‘Stronger Futures in the Northern Territory’ Bill [35] which calls for AMPs to be established by local NT communities, but they are required to meet minimum standards and to be approved by the Minister. These new AMPs will be reviewed in 2014. The current Queensland Government, building on the previous Government’s desire for an ‘exit strategy’ from AMPs, has promised to review them if violence and injury benchmarks are reached and women and children are not placed at greater threat.

## Discussion

It is of great social and historical significance for Indigenous Australians to understand how any reduction in alcohol-related injury and violence can be achieved, what it means in terms of changes in alcohol consumption, patterns of injuries, costs and benefits of any positive changes for individuals, families and communities and how they can be sustained. Surprisingly, any direct impacts and secondary consequences of Queensland’s highly controversial and historically significant reforms, which hold significant potential to address a key contributing risk factor to ‘close the gap’ in Indigenous health [24,36], have not been independently evaluated until now.

## Competing interests

All authors declare that they have no competing interests.

## Authors’ contributions

AC, supported by JR, ST and MF conducted consultations over several years to engage communities in the study. AC led the design and writing of the study protocol for funding, is the study manager, coordinates data collection, data management and data analysis. CW prepared and processed the Ethics applications, coordinated study implementation, data collection, data management, and data analysis and manuscript production. SM, CD, RM, AM, AS, VY, RS-F and DM conceived of the study and participated in its design. MF assisted to design the study component which examines drink driving offences and JR participated in the establishment of the study and the process evaluation study design, data collection and data management. All authors were involved in revising the manuscript for important intellectual content and read and approved the final manuscript.

## Pre-publication history

The pre-publication history for this paper can be accessed here:

http://www.biomedcentral.com/1471-2458/14/15/prepub
